# Chemical Insect Attractants Produced by Flowers of *Impatiens* spp. (Balsaminaceae) and List of Floral Visitors

**DOI:** 10.3390/ijms242417259

**Published:** 2023-12-08

**Authors:** Anna Jakubska-Busse, Izabela Czeluśniak, Marek Hojniak, Monika Myśliwy, Kamil Najberek

**Affiliations:** 1 University of Wroclaw, Faculty of Biological Sciences, Department of Botany, Kanonia 6/8, 50-328 Wroclaw, Poland; 2 University of Wroclaw, Faculty of Chemistry, F. Joliot-Curie 14, 50-383 Wroclaw, Poland; izabela.czelusniak@chem.uni.wroc.pl (I.C.); marek.hojniak@uwr.edu.pl (M.H.); 3 University of Szczecin, Institute of Marine and Environmental Sciences, Adama Mickiewicza 16, 70-383 Szczecin, Poland; monika.mysliwy@usz.edu.pl; 4 Institute of Nature Conservation, Polish Academy of Sciences, Al. Adama Mickiewicza 33, 31-120 Kraków, Poland; najberek@iop.krakow.pl

**Keywords:** alien species, balsams, chemical compounds, essential oils, floral extract, GC–MS, invasive

## Abstract

The study of the semiochemicals produced by the flowers of *Impatiens* spp. is an important topic that may explain the reason for the rapid expansion of some species in this genus. *Impatiens* L. belongs to the Balsaminaceae family, which includes several species considered to be invasive plants in Europe. This study aimed to characterize the phytochemistry of four naturally occurring plant species in Poland, including three invasive alien taxa (*Impatiens parviflora*, *I. glandulifera*, and *I. capensis*) and one native species (*I. noli-tangere*). Gas chromatographic techniques were used to assess phytochemical profiles of chemical attractant cues in their pollination biology. We detected differences in the scent profiles of the investigated species. All the examined *Impatiens* species produce various alcohols, i.e., heptacosanol, octacosanol, aldehydes (e.g., octadecanal, eicosanal, etc.), and fatty acids, as well as long-chain hydrocarbons such as dodecane, tricosane, petacosane, hexacosane, and farnesene. *Impatiens parviflora*, *I. glandulifera,* and *I. capensis* produce geraniol and linalool, which attract members of the Apidae family, including bumblebees and honeybees. *Impatiens parviflora* also produces linalool-derived monoterpenes (linalool oxide and 8-hydroxylinalool), which are a strong attractant for Diptera; this may clarify why the species is mainly visited and pollinated by syrphid flies. A list of insect visitors to the *Impatiens* species under study can be found in the article.

## 1. Introduction

The genus *Impatiens* and one species of the *Hydrocera* are classified to the Balsaminaceae family. The number of species within the genus depends on the author of the data, but the genus now includes more than 1000 species of flowering plants [1,2,3]. The family holds a special place among vascular plants, which are the most commonly studied group of alien species [4]. In Europe, there are four established alien species of the genus *Impatiens*: *I. capensis*, *I. balfourii*, *I. glandulifera,* and *I. parviflora,* and a single native species, *I. noli-tangere* [5]. The two former species are only invasive in specific regions of a few countries [6,7] whereas *I. glandulifera* and *I. parviflora* are prevalent pan-European species. *Impatiens glandulifera* has adverse effects on biological diversity, such as altering habitat structure and negatively impacting terrestrial gastropods [8]. The influence of *I. glandulifera* on vegetation depends on the initial species diversity of the patches. In species-rich plant communities, such as fresh meadows, a significant negative impact on plant species diversity was observed [9]. In species-poor patches, such as riparian tall herb vegetation, this impact is weak or even absent [10]. In contrast, the negative impact of *I. parviflora* on native flora and fauna is questioned; although the species penetrates new types of communities, it rarely becomes dominant [11]. At the same time, there are assumptions that it is a highly invasive species that may successfully compete with its native counterpart, *I. noli-tangere* [12]. In addition, *I. glandulifera* and *I. parviflora* may negatively impact crop pollination, because they may lure and co-opt pollinators (mainly bumblebees and hoverflies) that used to visit crop flowers (e.g., tomatoes and strawberries [13,14]). This can occur when *I. glandulifera* and *I. parviflora* occur in close proximity to crops, share the same pollinators, and have overlapping flowering periods. The impact of *I. capensis* on native plant species and communities is thought to be weaker compared to other non-native *Impatiens* species [15]. However, its presence in the secondary range poses ecological challenges, primarily arising from its high dispersion potential and its capacity to colonize habitats of significant conservation value [16]. Skálová et al. [17] suggest that it may outcompete the native *I. noli-tangere*, but to date, the species rarely co-occur. Furthermore, competition with native plants for pollinating insects is likely [18].

Nevertheless, it is still not clear which factors determine the invasive species’ success in new areas. To address this issue, researchers are conducting studies comparing invasive species with non-invasive ones. For example, it was demonstrated that *I. balfourii* and *I. glandulifera* exhibit similar reproductive, photosynthetic, and growth abilities, but only the latter species is widespread in Europe [19]. On the other hand, *I. glandulifera* seeds have a higher floating ability than those of *I. balfourii* [20]; in addition, *I. glandulifera* seeds are also less susceptible to attack by primary pathogens, resulting in a better seed performance of the invasive species [21]. Therefore, it is reasonable to consider that the success of *Impatiens* species is strongly associated with their seed traits. It should be stressed that the four alien *Impatiens* species are annual plants that disperse through seeds. At the same time, the successful development of *Impatiens* seeds is usually associated with pollinators visiting their flowers. The successful invasion of *Impatiens* therefore also depends on their floral traits, which enable them to attract insects. Plants use olfactory, visual, gustatory, tactile, and thermal stimuli signals, either individually or in combination [22,23]. The function of these signals is to ‘inform’ insects of the presence of nutritious rewards in flowers.

To date, the floral signals of European *Impatiens* species have been poorly investigated. The association between visitors and flower hue/area was tested for *I. glandulifera* and *I. parviflora* [13,24]. The floral reward offered by *I. glandulifera* (nectar volume and pollen protein content) was assessed under temperature and drought stress [25,26]. Furthermore, Wilson [27] explored the matching between bee pollinators and the flower morphology of *I. capensis*, while Bell et al. [28] used the floral gender of orange touch-me-nots as a cue.

In this study, we extended our understanding of floral cues in *Impatiens* species by investigating floral attractants that are strongly associated with successful insect attraction. This trait could play a crucial role in the success of the annual *Impatiens* species but has been poorly investigated to date. It is known that one of the key substances in the adaptation of plants to colonize new territories is the production of semiochemicals that influence the behavior of visiting and/or pollinating animals [29,30]. Repellent and attractant semiochemicals are substances emitted by plants, animals, and other organisms for chemically mediated communication [31]. These compounds are classified in terms of the responses they elicit from insects, as attractants, repellents, arrestants, deterrents, or stimulants [32,33]. Additionally, considering the communication level, semiochemicals are divided into allelochemicals and pheromones [34]. Allelochemicals are essential for interspecific communication, while pheromones are used for intraspecific communication [35]. The insect-attracting substances produced by plants may also include a number of compounds that may be associated with the waxes that coat the flowers, namely fatty alcohols, fatty acids, esters, and long-chain hydrocarbons [36]. These can also attract visiting insects, but only in close contact [37].

Plants produce allelochemicals to defend against attacks from their pests because emitting volatile organic compounds (VOCs) allows them to repel pests [38]. Additionally, they can attract natural enemies of those pests before or during their attack [38]. However, the production of substances that attract different groups of pollinators and repel phytophagous insects is of the greatest importance for the evolution and expansion of plants. Some invasive alien plants, such as *Fallopia baldschuanica* (Polygonaceae), can produce strong attractants and repellents that, in addition to rapid growth, ensure their evolutionary success [39].

*I. capensis*, and *I. glandulifera* secrete attractant semiochemicals to attract various pollinator groups, primarily bumblebees and bees (e.g., *Bombus pascuorum* and *Apis mellifera* [24,40,41]), which significantly increase their reproductive success. Although the success of *I. parviflora* is probably driven by its ability to self-pollinate autonomously [42], the species also benefits from its attractiveness to hoverflies (e.g., *Episyrphus balteatus*; [13,43]). Interestingly, alien *I. parviflora* and native *I. noli-tangere* share common pollinators (hoverflies, e.g., *E. balteatus*; [44]) and overlapping flowering periods. Although the latter species is pollinated mainly by long-tongue bumblebees (e.g., *B. hortorum* and *B. lapidarius*; [44]), it cannot be excluded that the potential attraction of hoverflies by *I. parviflora* may decrease the pollination rate of co-occurring native species. Although *I. noli-tangere*, like *I. capensis*, possesses the ability to produce cleistogamous flowers, which are closed and capable of self-pollination without the participation of external pollinators, the reproductive efficiency of such flowers is significantly lower. Typically, only 1-2(5) seeds form in the resulting fruits, whereas in capsules derived from open chasmogamous flowers, up to 9 seeds can develop [44].

The main aim of our work was to test (1) whether the *Impatiens* species studied differ in terms of the semiochemicals produced; (2) whether the expansion of *I. parviflora* and *I. glandulifera* observed in Europe may also be due to the production of more insect chemical attractants by these species; and (3) whether the observed floral morphological diversity in distinct *Impatiens* species correspond to variations in the semiochemicals they produce.

The presented results contribute new information about the biology of flowers of the *Impatiens* species established in Europe and may provide insights into the interactions between the species and pollinators that visit their flowers.

## 2. Results

### 2.1. GC-MS Analysis

To identify the organic compounds present in the nectars of four tested *Impatiens* species, dichloromethane extracts from the flowers containing nectar were analysed by GC/MS chromatography (Table 1). As expected, the composition of the extracts of all tested species differed from each other. The *I. parviflora*, *I. glandulifera,* and *I. capensis* extracts contain more chemical compounds than *I. noli-tangere* extracts.

In the samples of *I. parviflora* floral extracts, 44 chemical compounds were identified (Table 1). The oxygen-containing compounds were dominated by aliphatic alcohols (e.g., linalool, heptacosanol, docosanol, etc.) and aldehydes (e.g., pelargonaldehyde, docosanal, etc.). In addition to linalool, two of its derivatives, linalool oxide and 8-hydroxylinalool, were also present in the samples. Among the aromatic compounds, phenylmethanol (benzyl alcohol) and ethyl-4-etoxybenzoate were found. The extracts also contained five saturated fatty acids (lauric, myristic, palmitic, stearic, and arachic) and one unsaturated fatty acid—linoleic acid. The presence of aliphatic hydrocarbons, both linear and branched, was also detected in *I. parviflora* extracts.

**Figure 1:**
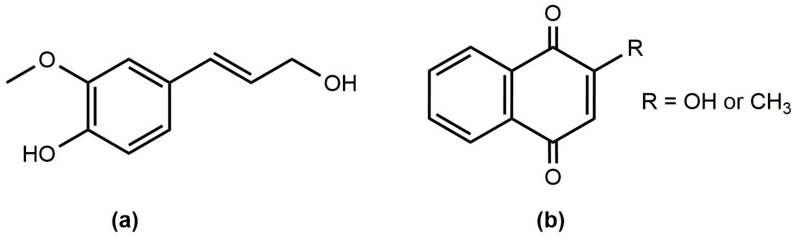
Chemical structure of some identified compounds: (**a**) conipheryl alcohol; and (**b**) naphthoquinone derivatives.

A comparable hydrocarbon composition was characterized in samples of *I. glandulifera* flower extracts (Table 1). In addition to pentacos-1-ene, which is present in both species, hexacos-1-ene was also identified in *I. glandulifera* flower extracts. Similar to *I. parviflora*, the oxygen-containing compounds were dominated by aliphatic components. However, in addition to phenylmethanol, phenylethanol and conipheryl alcohol (Figure 1a) were also detected. The composition of fatty acids also included capric acid and ethyl docosanoate.

The most oxygen-containing compounds were detected in *I. capensis* flower extracts (Table 1). Most of these were also present in the *I. parviflora* and *I. glandulifera* species. In addition, heptadecan-2-one, 1-eicosanol, and two aldehydes; 1-heptadecanal and 1-hexacosanal, were identified in the extracts of *I. capensis* flowers. The composition of fatty acids and long-chain carbohydrates was similar to that of the above-mentioned species.

Although the content of the compounds identified in the extracts of *I. noli-tangere* was lower, many compounds known as attractants for pollinators, such as nonanal, phenylethanol, and nonan-2-one, were detected (Table 1). In addition, three fatty acids (palmitic, linolenic, and arachic), various branched and linear-chain hydrocarbons (e.g., hexadecane, octadecane, tricosane, and nonacosane) were common among the extracts of all tested species. These also included unsaturated hydrocarbons such as farnesene, pentacos-1-ene, heptacos-1-ene, etc.

In the extracts of all investigated *Impatiens* species, two flavonoids, 1,4-naphtalenedione 2-hydroxy and 1,4-naphtalenedione 2-metoxy, were detected (Figure 1b). However, both naphthoquinone derivatives were recorded only in the cases of *I. glandulifera* and *I. noli-tangere*.

### 2.2. Flower Visitors

This study showed that the visiting insects observed on the flowers of the four *Impatiens* species differ in their food preferences. We observed both hoverflies, insects that feed on pollen, and those insects that feed on the nectar produced in the flower spur, mainly honeybee and bumblebee species. Although the populations of the different *Impatiens* species studied were located in different parts of Poland, similar groups of insects were observed visiting the flowers (Table 2).

**Figure 2:**
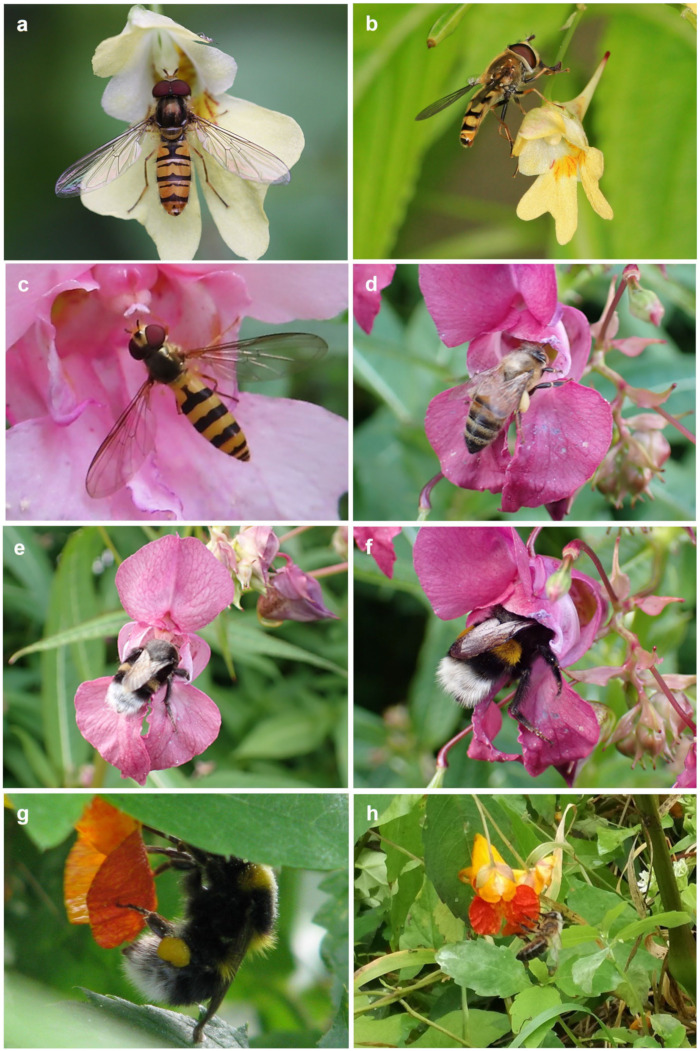
Insect visitors to flowers of *Impatiens* species: (**a**) *Episyrphus balteatus* on *Impatiens parviflora*; (**b**) *Eupeodes corollae* on *I. parviflora*; (**c**) *E. corollae* on *I. glandulifera*; (**d**) *Apis mellifera* on *I. glandulifera*; (**e**,**f**) *Bombus terrestris* on *I. glandulifera*; (**g**) *B. hortorum* on *I. capensis*; and (**h**) *A. mellifera* on *I. capensis*.

Himalayan balsam (*I. glandulifera*) was most frequently visited by hymenopterans, which we observed as flower visitors (Figure 2d–f, Table 2). Flowers of this species mainly attracted both bees and bumblebees. We often recorded holes in the spur, indicating feeding by nectar thieves (most likely bumblebees). The hoverflies *Episyrphus balteatus* and *Eupeodes corollae* (Diptera, Syrphidae), which feed on pollen and nectar, were also frequent visitors to this invasive plant. Occasionally we also observed another hoverfly *Helophilus trivittatus*, and the hummingbird hawkmoth *Macroglossum stellatarum*, as well as the common wasp *Vespula vulgaris*, and seven-spotted ladybug *Coccinella septempunctata* visiting flowers.

Orange balsam (*I. capensis*) was visited by Hymenoptera, representatives of Apoidea, i.e., *Bombus* species (Figure 2g) and honeybee *Apis mellifera* (Figure 2h), as well as (though rarely) Halictid species. The flowers of this species are also attractive to representatives of the syrphid fly *E. balteatus* and *V. vulgaris* (Table 2).

The flowers of *I. noli-tangere* were visited by Hymenoptera from the genera *Bombus*, *Apis*, *Lasioglossum* sp. and flies (Diptera), representatives of the Muscidae and Syrphidae families (Table 2). It should be noted, however, that these visits were not as frequent as visits to *I. glandulifera* or *I. capensis*.

Surprisingly, we observed mainly Syrphidae feeding on *I. parviflora* flowers in all populations studied (Figure 2a,b and Table 2). The common carder bee *B. pascuorum* and adult ladybirds (*C. septempunctata*) were occasionally observed feeding on the flowers of this species.

## 3. Discussion

It is well known that the flowers of the *Impatiens* have enormous diversity and a variety of pollinators; the species of flower-visiting insects observed depend on the different climatic regions in which the plants grow [45]. According to the literature, *Impatiens* species are generally pollinated by bumblebees, hummingbirds, and butterflies [46,47,48,49]. In central Europe, the main pollinators of this genus are bees (Apidae) and flies (Diptera) [44], which we often observed.

The different *Impatiens* species we studied were visited by essentially the same groups of potential pollinators, i.e., *Apis mellifera* and *Bombus* spp. Surprisingly, *I. parviflora* was unique in this regard, as we recorded mainly syrphid flies visiting its flowers, while bumblebees were recorded rarely. It could be a result of the small size of the flowers produced by this alien species, which is not preferred by bumblebees. This may also be due to the fact that *I. parviflora* grows in habitats where bumblebee species are rare foragers and/or this invasive plant does not produce enough nectar to attract the interest of bumblebees.

Until now, it was not clear how the studied *Impatiens* species attract pollinating insects using chemical compounds. Chemical attractants of *Impatiens* species have not been studied to date, and, consequently, their potential role in the pollination of *Impatience* spp. remains unknown.

The species studied differ in the color and size of their flowers, which may be important in pollination biology. Experimental evidence has previously confirmed that *I. glandulifera* attracts a significantly higher number of pollinators compared to native plants, impacting the reproductive success of the indigenous flora [50]. Certainly, *I. glandulifera* produces the largest flowers of the species studied and is most frequently visited by bumblebees, which was confirmed by our observations (Figure 2e,f). It should be noted that this species has flowers with the most intense color among those examined. Indeed, two quinone pigments, lawsone and 1,4-naphtalenedione 2-metoxy, were found in the samples of *I. glandulifera* floral extract. Furthermore, Lobstein et al. [51] found that the naphthoquinone content of the flowering aerial part of *I. glandulifera* was significantly higher than the content in three other species. The two naphthoquinones have been known to possess anti-fungal properties [52]. Interestingly, the *Helophilus trivittatus* we identified as a visitor to *I. glandulifera* is a pollen- and nectar-feeding fly that is known to visit mainly yellow and purple flowers [53].

As expected, all the studied species produced strong chemical attractants that are likely to have a role in attracting pollinators. Among the identified compounds, several are known from the literature to act as strong lures for certain groups of insects.

The tested species produce fatty acids such as palmitic, linoleic, stearic, and eicosanoic (arachic) acids, compounds that are attractants for many groups of insects, both Diptera and Hymenoptera, including *B. terrestris* [54,55]. Palmitic, lauric, and stearic acids have also been found to be strong attractants for the honeybee *A. mellifera* [56,57].

They also produce long-chain hydrocarbons, such as dodecane, tricosane, pentacosane, and hexacosane, which are known in the literature to attract bees (*Andrena* sp.) and bumblebees [58,59]. Interestingly, compounds such as tricosane, pentacosane, hexacosane, octacosane, nonacosane and hentriacontane are also attractants of wasps [55], which, in our study, were seen to have visited *I. glandulifera* and *I. capensis*.

Oxygen-containing compounds found in the samples of investigated species, such as nonane-2-one and aldehydes: octadecanal, eicosanal, docosanal, and tetracosanal, act as strong lures for many groups of insects including Hymenoptera, Andrenidae [60] and Apidae [57], and mainly *Bombus* spp. [60]. However, the alcohols, pentacosanol, heptacosanol, and octacosanol, are recognized as an attractant only for Hymenoptera, Apidae, and the tribe Meliponini, which includes, for example, the African stingless bee (*Hypotrigona* species) [55,61].

Surprisingly, the monoterpene alcohols, linalool and geraniol, were only detected in invasive *Impatiens* species such as *I. parviflora*, *I. glandulifera,* and *I. capensis*. In the studied population of native *I. noli-tangere*, these compounds were not found.

Linalool is important in nature as a key compound in the complex pollination biology of various plant species to ensure reproduction [62]. This monoterpene has a specific olfactory description: “light and refreshing, floral-woody, with a faint citrus note” [63] and is produced by many plant species belonging to different botanical families, including Lamiaceae, Lauraceae, and Verbenaceae [64]. It is known to attract a wide range of pollinators (e.g., bees and butterflies), herbivores, and parasitoids [64]. Due to its properties, linalool is also used as a natural repellent against various insects that damage crops [62]. Data from the literature confirm the toxicity of this monoterpene alcohol in a dose-dependent manner against the beetles *Tribolium castaneum* and *Oryzaephilus surinamensis* [65].

Geraniol, like linalool, is a strong bee attractant [66]. This unsaturated aliphatic alcohol usually appears as a clear liquid with a sweet, floral odor. It is a major constituent of the essential oil of damask rose and has also been found in the essential oils of tea, lemongrass (*Cymbopogon flexuosus*), lavender, plum, and grape [67]. Honeybee olfactory glands produce geraniol to mark nectar-bearing flowers and locate hive entrances [68] to attract flesh flies (Sarcophagidae) and braconid wasps [69].

The presence of linalool and geraniol along with the above-mentioned compounds in the scent profile may explain the frequent visits of honeybees to flowers that we observed (Figure 2d,h). The emissions of these substances could reflect an adaptation of *I. glandulifera* facilitating its expansion, as they are documented as effective attractants for pollinators.

In addition, samples of *I. glandulifera* floral extract contained the conipheryl alcohol, commonly known as an attractant not only for queens of *A. mellifera* [70] but also for *Bactrocera* fruit flies [55,71]. Thus, the presence of this alcohol in the nectar might suggest more frequent visits of the flies to the flowers of this species.

Among the species studied, the flowers of *I. parviflora* were most frequently visited by members of the Syrphidae of the Diptera. This may be due to the small size of the flowers produced, which are too small for most bumblebee species to land on. On the other hand, we believe that it is also due to the fact that this species produces linalool-related monoterpenes (linalool oxide and 8-hydroxylinalool), which, together with linalool, are known to attract Diptera [72,73,74].

It should also be added that farnesene, which occurs in other species, was found in samples of this species and is known in the literature to be a strong attractant for members of Diptera. This may also explain why *I. parviflora* was frequently visited by hoverflies. Studies in the literature confirmed the key role of these chemicals in attracting some aphid predators and parasitoids, including ladybeetles and syrphid flies [39,75,76].

Interestingly, we occasionally observed *Coccinella septempunctata* (Coleoptera) on *I. parviflora* and *I. glandulifera* flowers; its presence may be related to a reaction to farnesene. This compound is known in the literature not only for its antibacterial, antifungal, and sedative properties, but also shows its strongest effects as an alarm pheromone [39]. It is also possible that the presence of ladybugs was related to aphids that appeared on other plant species growing nearby.

*I. parviflora* belongs to the invasive and expansive species, in contrast to *I. noli-tangere*, which is a naturally occurring species in Europe. Under natural conditions, these species often co-occur, and their flowering phases overlap, which may explain the observations of *A. mellifera*, *Bombus* sp., and the syrphid flies visiting the flowers of both *I. parviflora* and *I. noli-tangere* in neighboring populations of both species. In light of the results obtained, it seems that *I. parviflora* produces stronger insect attractants (e.g., phenylmethanol, geraniol, heptan-2-one, 1-tetradecene, 1-docosanol, linalool, and linalool oxide) than its native counterpart. Therefore, it cannot be ruled out that *I. parviflora* may reduce the pollination rates of *I. noli-tangere* by attracting hoverflies that have also been reported to visit the native balsam [44].

Plant volatiles can be used to synergize and enhance the attractiveness of insect pheromones. They form the basis of highly attractive baits but can also act as a feeding deterrent or as a repellent signal to potential pests [77].

In light of the results obtained, it seems that one of the factors affecting the success of the invasion may also be the production of a large number of chemical attractants by *Impatiens* species.

## 4. Materials and Methods

### 4.1. Plant Material

Fresh flowers with visible nectar secretion of investigated *Impatiens* species used for the chemical analyses were collected from natural populations of analyzed species, including: *I. parviflora* from populations located in the vicinity of Wrocław (51°07′24″ N 17°05′41″ E), from Wałkowa near Milicz (GPS 51°30′08″ N 17°18′47″ E), Henryków near Ziębice (GPS 50°39′32″ N 17°01′52″ E) and from individuals occurring in Krakow and neighboring Marcyporaba (GPS 49°55′19.7″ N 19°37′38.0″ E) between 24 July 2020 and 29 August 2023. Fresh flowers for the scent analysis of *I. glandulifera* were collected from populations located in Wrocław (51°05′39″ N 17°05′40″ E), Bystrzyca Kłodzka (GPS 50°18′20″ N 16°39′16″ E), and also from populations localized in the vicinity of Kraków, Marcyporęba and Ochodza (GPS GPS 49°58′36.9″ N 19°44′59.7″ E). *I. noli-tangere* flowers were collected from populations in Wrocław-Rędzin (51°10′56″ N 16°56′16″ E), and Starczów near Ziębice (GPS 50°33′50″ N 16°56′23″ E), while the flowers of *I. capensis*, due to the limited distribution area of this species in Poland, were collected from four closely situated populations in Western Pomerania: Police (GPS 53°33′28.4″ N 14°34′14.2″ E), Szczecin-Zdroje (GPS 53°23′09.9″ N 14°37′09.5″ E), Załom (GPS 53°26′31″ N 14°42′20.7″ E), and Lubczyńskie Łęgi (GPS 53°29′20.9″ N 14°41′40.2″ E).

### 4.2. Field Observations of Insect Activity

Observations were made to determine which groups of insects visit and pollinate *Impatiens* flowers. These data were needed to verify the results of the chemical analyses. The field observations were conducted once per each locality and included recording the number of insects, the types of resources collected (nectar, pollen), and the time the flowers were handled (to determine whether visiting insects were not random). Observations were carried out occasionally from 2 July to 15 September 2020–2023 in the above-mentioned populations, situated in different parts of Poland: Lower Silesia (SW Poland), Kraków (Lesser Poland), and Western Pomerania (NW Poland). The locations where insect behavior was studied were the same as those where the samples for chemical analysis were collected. Their GPS coordinates are given in Section 4.1. Observations were made over a span of 2–6 h, covering daylight hours (9:00 a.m.–6:00 p.m.). The visitor insects were photographed/documented using a Nikon D50 camera with a Tamron 90 mm f/2.8 SP Di Macro lens, captured in field conditions by A.J.-B. and identified by specialists. Bumblebees, as legally protected insects in Poland, were not caught but instead identified on the basis of macrophotography.

### 4.3. GC/MS Analysis of Nectar Composition

The flowers of *I. parviflora* (n = 350), *I. glandulifera* (n = 300), *I. noli-tangere* (n = 250), and *I. capensis* (n = 250) were collected. Prepared samples of 30–50 flowers containing nectar (depending on their size), were collected in 5 mL glass vials followed by the addition of dichloromethane (Sigma-Aldrich, Merck Life Science, Poznan, Poland, 99.9%) at room temperature. The dichloromethane (1–2 mL) was used to extract foliar nectar drops. Finally, approximately 0.5 mL of the floral extract was obtained for each sample. The extracts were stored at −15 °C until used for GC/MS analyses. Seven samples of the extract of *I. parviflora* flowers, 9 samples of *I. glandulifera*, 5 samples of *I. noli-tangere,* and 9 samples of *I. capensis* were prepared and analyzed by means of GC/MS chromatography. Chemical analyses were conducted during two research seasons: 2022 and 2023. Only compounds detected in all or the majority of samples from the same species are included in Table 1.

GC/MS chromatography was performed on a GCMS-QP2010SE Shimadzu gas chromatograph mass spectrometer (MS scan 35–600 *m*/*z*) and Zebron ZB-5ms (30 m 0.25 mm; Phenomenex) column. The oven temperature at the start of the measurement was 40 °C, and then increased at a rate of 4 °C/min until it reached 120 °C. The oven temperature was increased to 320 °C at a rate of 40 °C/min, then kept at 320 °C for 5 min. The injection port temperature was 250 °C. Helium (1.2 mL/min) was used as a carrier gas. A total of 1 μL of each extract was injected using the splitless technique.

Identification of the compounds was carried out using the NIST17 database. For the identification of long-chain hydrocarbons, samples of C8–C36 alkanes were analyzed by GC/MS using the same oven and column parameters; their spectra and retention times were compared with those obtained in the extracts.

## 5. Conclusions

In the floral extracts of the *Impatiens* species studied, the presence of attractants for bees was detected, including alcohols (e.g., pentacosanol, heptacosanol and octacosanol) and aldehydes (e.g., peralgonaldehyde, octadecanal, eicosanal, etc.), as well as long-chain hydrocarbons (e.g., dodecane, tricosane, petacosane, farnesene, etc.) and fatty acids (palmitic, lauric, stearic, etc.). In addition, *I. parviflora*, *I. glandulifera*, and *I. capensis* also produce geraniol and linalool, which attract bumblebees and honeybees. Field observations of the activity of these insect groups, combined with extensive reports on chemical attractants that attract them, suggest that floral chemical compounds could play an important role in the population biology of these invasive species. Based on the results obtained, we hypothesize that the chemical composition of the floral scent of non-native *Impatiens* spp. may be a key factor in the success of these species in the European secondary range, as the chemicals attract a large group of local pollinators. As a result, the alien balsams may outcompete native plants for pollinators. This scenario could occur in competition with a single native European balsam, *I. noli-tangere*, which shares similar habitat preferences and common pollinators with its alien counterparts.

## Figures and Tables

**Table 1:** List of organic compounds identified in floral extracts of investigated *Impatiens* species.

No	Name	Chemical Formula	CAS No	IPar	IGlan	INol	ICap
Oxygen-containing compounds							
1	heptan-2-one	C_7_H_14_O	110-43-0	A	-	-	-
2	phenylmethanol	C_7_H_8_O	100-51-6	A	B	-	A
3	phenylethanol	C_8_H_10_O	60-12-8	-	B	-	A
4	nonan-2-one	C_9_H_18_O	821-55-6	A	A	B	B
5	pelargonaldehyde (nonanal)	C_9_H_18_O	124-19-6	A	-	B	B
6	*p*-vinylguaiacol (2-methoxy-4-vinylphenol)	C_9_H_10_O_2_	7786-61-0	A	A	-	A
7	conipheryl alcohol	C_10_H_12_O_3_	458-35-5	-	B	-	-
8	geraniol	C_10_H_18_O	106-24-1	B	B	-	A
9	linalool (2,6-dimethyl-2,7-octadien-6-ol)	C_10_H_18_O	78-70-6	A	A	-	A
10	linalool oxide (trans-tetrahydro-2,2,6-trimethyl-6-vinyl-2H-pyran-3-ol)	C_10_H_18_O_2_	39028-58-5	A	-	-	-
11	8-hydroxylinalool (2,6-dimethyl-2,7-octadiene-1,6-diol)	C_10_H_18_O_2_	64142-78-5	A	-	-	-
12	ethyl 4-ethoxybenzoate	C_11_H_14_O_3_	23676-09-7	A	-	A	B
13	1-heptadecanal	C_17_H_34_O	629-90-3	-	-	-	B
14	heptadecan-2-one	C_17_H_34_O	2922-51-2	-	-	-	B
15	octadecanal	C_18_H_36_O	638-66-4	A	A	-	A
16	nonadecan-2-one	C_19_H_38_O	629-66-3	A	A	B	A
17	eicosanal	C_20_H_40_O	2400-66-0	A	B	-	B
18	1-eicosanol	C_20_H_40_O	629-96-9	-	-	-	A
19	1-docosanal	C_22_H_46_O	57402-36-5	A	B	A	A
20	1-docosanol	C_22_H_46_O	661-19-8	A	A	-	A
21	1-tetracosanol	C_24_H_50_O	506-51-4	-	-	-	A
22	1-tetracosanal	C_24_H_48_O	57866-08-7	B	-	A	B
23	1-pentacosanol	C_25_H_52_O	26040-98-2	A	B	-	A
24	1-hexacosanal	C_26_H_52_O	26627-85-0	-	-	A	A
25	1-hexacosanol	C_26_H_54_O	506-52-5	-	-	A	-
26	1-heptacosanol	C_27_H_56_O	2004-39-9	A	A	A	A
27	1-octacosanal	C_28_H_56_O	22725-64-0	-	-	A	A
28	1-octacosanol	C_28_H_58_O	557-61-9	A	A	A	A
**Fatty acids and their esters**							
29	decanoic (capric) acid	C_10_H_20_O_2_	334-48-5	-	B	-	-
30	tetradecanoic (myristic) acid	C_12_H_28_O_2_	544-63-8	B	A	-	-
31	dodecanoic (lauric) acid	C_16_H_32_O_2_	59154-43-7	A	A	-	B
32	hexadecanoic (palmitic) acid	C_16_H_32_O_2_	57-10-3	A	A	A	A
33	9,12,15-octadecatrienoic (linolenic) acid	C_18_H_30_O_2_	463-40-1	A	A	A	A
34	octadecanoic (stearic) acid	C_18_H_36_O_2_	57-11-4	A	A	-	A
35	eicosanoic (arachic) acid	C_20_H_40_O_2_	506-30-9	B	A	A	A
36	ethyl docosanoate	C_24_H_48_O_2_	5908-87-2	-	A	-	B
37	methyl tetracosanoate	C_25_H_50_O_2_	2442-49-1	-	-	A	B
**Long-chain hydrocarbons**							
38	undecane	C_11_H_24_	1120-21-4	A	A	A	A
39	dodecane	C_12_H_26_	112-40-3	A	A	-	-
40	tetradecane	C_14_H_30_	629-59-4	A	A	-	A
41	tetradec-1-ene	C_14_H_28_	1120-36-1	B	A	-	B
42	farnesene	C_15_H_24_	18794-84-8	A	B	B	-
43	hexadecane	C_16_H_34_	544-76-3	A	A	B	A
44	heptadecane	C_17_H_36_	629-78-7	A	A	A	A
45	octadecane	C_18_H_38_	593-45-3	A	A	A	A
46	nonadecane	C_19_H_40_	629-92-5	A	A	-	A
47	eicosane	C_20_H_42_	112-95-8	A	A	-	A
48	neophytadiene	C_28_H_38_	504-96-1	B	B	B	B
49	heneicosane	C_21_H_44_	629-94-7	A	A	A	A
50	docosane	C_22_H_46_	629-97-0	A	A	A	A
51	tricosane	C_23_H_48_	638-67-5	A	A	A	A
52	pentacosane	C_25_H_52_	629-99-2	A	A	A	A
53	pentacos-1-ene	C_25_H_50_	16980-85-1	A	A	-	A
54	hexacosane	C_26_H_54_	630-01-3	-	-	-	A
55	hexacos-1-ene	C_26_H_52_	18835-33-1	-	A	A	B
56	heptacosane	C_27_H_56_	593-49-7	A	B	A	A
57	heptacos-1-ene	C_27_H_54_	15306-27-1	-	-	A	-
58	octacosane	C_28_H_58_	630-02-4	-	-	-	A
59	nonacosane	C_29_H_60_	630-03-5	A	A	A	A
60	triacontane	C_30_H_62_	638-68-6	-	-	-	A
61	hentriacontane	C_31_H_64_	630-04-6	A	A	A	A
**Flower pigments**							
62	1,4-naphtalenedione 2-hydroxy (lawsone)	C_10_H_6_O_3_	83-72-7	A	A	A	A
63	1,4-naphtalenedione 2-metoxy	C_11_H_8_O_3_	2348-82-5	-	A	A	-

Abbreviations: IPar—*I. parviflora*, IGlan—*I. glandulifera*, INol—*I. noli-tangere*, ICap—*I. capensis*; + compounds present, - compounds not detected; number of compound replicates: A—all samples, B—majority of samples.

**Table 2:** List of insects visiting the studied *Impatiens* species.

Insect Order	Family	Species	Flower Visitation Rate				Type of Floral Reward
			IPar	IGlan	INol	ICap	
**Hymenoptera**	Apidae	*Apis mellifera*	C	A	B	A	n, p
		*Bombus* sp.	C	A	B	A	n, p
		*Bombus hortorum*	-	-	B	C	n, p
		*Bombus hypnorum*	-	B	-	-	n, p
		*Bombus lucorum*-complex (including *B. lucorum*, *B. cryptarum* and *B. magnus*)	-	B	-	-	n, p
		*Bombus pascuorum*	C	A	B	A	n, p
		*Bombus terrestris*	-	B	B	-	n, p
	Vespidae	*Vespula vulgaris*	-	C	-	B	n
	Halictidae	*Halictus* sp.	-	-	-	C	p
		*Lasioglossum* sp.	-	B	C	C	n
**Diptera**	Muscidae	*Musca domestica*	-	-	C	-	p
	Syrphidae	*Melanostoma* sp.	C	-	C	-	n *, p
		*Eupeodes corollae*	A	B	-	-	n *, p
		*Episyrphus balteatus*	A	B	B	B	n *, p
		*Helophilus trivittatus*	-	C	-	-	p
		*Sphaerophoria scripta*	C	-	C	-	n *, p
		*Syrphus ribesii*	B	-	B	-	n *, p
**Lepidoptera**	Sphingidae	*Macroglossum stellatarum*	-	C	-	-	n
**Coleoptera**	Coccinellidae	*Coccinella septempunctata*	C	C	-	-	n

Abbreviations: IPar—*I. parviflora*, IGlan—*I. glandulifera*, INol—*I. noli-tangere*, ICap—*I. capensis*; - Insect species not observed; Insect visitation rates: A—very often, B—often, C—rare; Types of resources collected by the visitors: n—nectar, p—pollen; * Syrphids have the ability to collect nectar exclusively from the flowers of *I. parviflora*.

## Data Availability

Data are contained within the article.
